# Modular Organization of Signal Transmission in Primate Somatosensory Cortex

**DOI:** 10.3389/fnana.2022.915238

**Published:** 2022-07-08

**Authors:** Yaqub Mir, László Zalányi, Emese Pálfi, Mária Ashaber, Anna W. Roe, Robert M. Friedman, László Négyessy

**Affiliations:** ^1^Theoretical Neuroscience and Complex Systems Group, Department of Computational Sciences, Wigner Research Centre for Physics, Budapest, Hungary; ^2^János Szentágothai Doctoral School of Neurosciences, Semmelweis University, Budapest, Hungary; ^3^Department of Anatomy, Histology and Embryology, Semmelweis University, Budapest, Hungary; ^4^California Institute of Technology, Department of Biology and Biological Engineering, Pasadena, CA, United States; ^5^Division of Neuroscience, Oregon National Primate Research Center, Oregon Health and Science University, Beaverton, OR, United States; ^6^Interdisciplinary Institute of Neuroscience and Technology, School of Medicine, Zhejiang University, Hangzhou, China; ^7^Key Laboratory of Biomedical Engineering of Ministry of Education, College of Biomedical Engineering and Instrument Science, Zhejiang University, Hangzhou, China

**Keywords:** anterograde labeling, bouton, convergence, multivariate analysis, squirrel monkey

## Abstract

Axonal patches are known as the major sites of synaptic connections in the cerebral cortex of higher order mammals. However, the functional role of these patches is highly debated. Patches are formed by populations of nearby neurons in a topographic manner and are recognized as the termination fields of long-distance lateral connections within and between cortical areas. In addition, axons form numerous boutons that lie outside the patches, whose function is also unknown. To better understand the functional roles of these two distinct populations of boutons, we compared individual and collective morphological features of axons within and outside the patches of intra-areal, feedforward, and feedback pathways by way of tract tracing in the somatosensory cortex of New World monkeys. We found that, with the exception of tortuosity, which is an invariant property, bouton spacing and axonal convergence properties differ significantly between axons within patch and no-patch domains. Principal component analyses corroborated the clustering of axons according to patch formation without any additional effect by the type of pathway or laminar distribution. Stepwise logistic regression identified convergence and bouton density as the best predictors of patch formation. These findings support that patches are specific sites of axonal convergence that promote the synchronous activity of neuronal populations. On the other hand, no-patch domains could form a neuroanatomical substrate to diversify the responses of cortical neurons.

## Introduction

Cortical connectivity at the neuronal population or mesoscale level exhibits a strong structure–function relationship, as shown in the primate somatosensory cortex (Wang et al., 2013). It is well known that long-range cortico-cortical axonal connections of nearby pyramidal neurons form a patchy pattern (for reviews, see Douglas and Martin, 2004; Rockland, 2020). Likewise, cortical microstimulation results in activation patterns similar to the distribution of lateral connectivity (Roe et al., 2015; Xu et al., 2019; Friedman et al., 2020). The similarity of the response properties of neurons in axonal patches with their parent soma supports their functional significance, as seen in the case of orientation columns in the primary visual cortex (Malach et al., 1993; Kisvárday et al., 1997). In the primate somatosensory cortex, tactile modalities are mapped in a columnar manner, similar to orientation domains in the visual cortex (Mountcastle, 1997; Friedman et al., 2004). Similar patchy organization of lateral connectivity in the visual and somatosensory cortex suggests that it plays a fundamental role in cortical processing in primates (Lund et al., 1993).

At the population level, the axonal patches of pyramidal neurons are formed by recurrent collaterals bearing high-density bouton clusters (Buzás et al., 2006; Binzegger et al., 2007; Muir and Douglas, 2011). These distal clusters are connected to the parent soma by linear axonal segments with boutons that can establish rich connectivity (Malach et al., 1993; Kisvárday et al., 1997; Buzás et al., 2006). Patches are not organized exclusively by functional preference, as shown for the orientation circuitry of the primary visual cortex, but there is a bias in their distribution toward the formation of like-to-like connectivity (Kisvárday, 2016). In addition, bouton-forming linear axonal segments cross an array of neuronal populations with diverse tuning properties (see figures in Buzás et al., 2006; Martin et al., 2014). Understanding the mechanisms of specificity and diversity of neuronal connections is a central question in deciphering cortical computation. How information propagated by axonal patches and no-patch linear axonal segments contribute to cortical computation is not known.

The morphological properties of axons, such as thickness, tortuosity, bouton spacing, and myelination, can influence signal transmission in various ways, such as conduction velocity (delay) and synaptic integration via single or multiple synapses (Segev and Schneidman, 1999; Anderson et al., 2002; Shepherd et al., 2002; Angelucci and Bressloff, 2006; Binzegger et al., 2007; Innocenti, 2017; Koestinger et al., 2017). In addition to individual axonal properties, the collective nature, i.e., the convergent and divergent characteristics, of bouton-forming axonal branches also plays a crucial role in cortical functioning by supporting synchronization or providing redundancy (Douglas and Martin, 2007; Muller et al., 2018). Based on these observations, the major goal of this study was to unravel the computational role of unmyelinated bouton-forming axons of the gray matter within and outside the patches using tract tracing and quantitative morphological approaches. Specifically, we aimed to identify individual and collective axonal properties that distinguish the role of patch and no-patch axonal connections in cortical function. We tested the hypothesis that patches are formed by the convergence of axonal branches with high bouton densities whereas no-patch axons have low bouton densities and are not convergent. We studied intrinsic and interareal connections in somatosensory cortical areas 3b and 1 of the squirrel monkey, where, in line with earlier observations (Krubitzer and Kaas, 1990; Lund et al., 1993; Manger et al., 1997), we described a patchy pattern of axonal labeling (Négyessy et al., 2013; Ashaber et al., 2014, 2020; Pálfi et al., 2018). In addition, we described a dense array of straight, bouton-forming axonal segments, which urged further investigations regarding the role of these axonal segments in intrinsic and interareal connectivity and cortical processing. The interconnection of these somatosensory areas allowed us to compare feedforward and feedback patch and no-patch domains in addition to that of the intrinsic connections as areas 3b and 1 are thought to have a hierarchical organization (Kaas, 1983, 2004; Iwamura, 1998; Rossi-Pool et al., 2021).

## Materials and Methods

### Tracer Injection and Tissue Processing

Animal care and surgical procedures were performed in compliance with the National Institutes of Health (NIH) regulations and with the approval of the Institutional Animal Care and Use Committee of Vanderbilt University. Three male and three female adult squirrel monkeys (Saimiri sciureus weighing 600–800 g, 2–9 years old) were used, which were subjects in our prior studies on the connectivity of distal finger pad representations of areas 3b and 1 (Négyessy et al., 2013; Ashaber et al., 2014, 2020; Pálfi et al., 2018). Here, we provide a brief description of the experimental procedures (for details, see Négyessy et al., 2013; Ashaber et al., 2014).

As previously described, each animal was sedated (ketamine, 15 mg/kg, im), placed in a stereotaxic frame, mechanically ventilated with isoflurane anesthesia, and hydrated with lactated ringers via intravenous infusion. Analgesia during surgery was supplied by buprenorphine (0.01 mg/kg, im). Vital signs [peripheral capillary oxygen saturation, heart rate, electrocardiogram (ECG), end-tidal carbon dioxide, respiratory pattern, and temperature] were monitored. After craniotomy [centered at AP 6 mm and ML 15 mm (Gergen and MacLean, 1962)] and durotomy, areas 3b and 1 were located using the central sulcus and blood vessel landmarks. Following the electrophysiological mapping of hand and finger representations, intrinsic signal optical imaging was used to identify the distal finger pad representations of fingers D2–D4 in areas 3b and 1. Biotinylated dextran amine (BDA, 1:1 mixture of 10% 10K and 10% 3K, Molecular Probes, Inc.) in 0.01 M phosphate buffer (PB, pH 7.4) was then injected into a distal finger pad representation in either area 3b or 1 via iontophoresis (3 μA, 7 s on/off cycle for 20 min) (three cases each). In all cases, the core (≤ 300 μm in diameter) of the BDA injection uptake zone included the upper layers (200–390 μm below the surface), while the lower cortical layers were also included in the core in cases of area 1 injections (160–800 μm below the surface) [for more details, see Table 1 in Négyessy et al. (2013) and Ashaber et al. (2014)]. Upon recovery, heat support was provided for the first 12 h with postoperative analgesia supplied by buprenorphine (0.01 mg/kg, im, two times a day) for 3 days. After a 10–20-day survival period, animals were deeply anesthetized before being transcardially perfused with a fixative composed of 4% paraformaldehyde, 0.1% glutaraldehyde, and 0.2% picric acid in 0.1 M PB (pH 7.3). The brains were immediately removed, and the region of interest was then cut from the cortex, flattened parallel to the cortical surface, and postfixed overnight in 4% paraformaldehyde.

**Table 1:** Total number of reconstructed axons labeled by area 3b injection in the different studied pathways.

**Injected area**	**Area 3b**								
	**P**				**NP**				
	**FF**		**Intra**		**FF**		**Intra**		**Total**
**Cases**	**Supra**	**Infra**	**Supra**	**Infra**	**Supra**	**Infra**	**Supra**	**Infra**	
J	6	3	6	3	6	3	6	3	36
Mac	3	3	3	3	3	3	3	3	24
V	3	3	3	3	3	3	3	3	24
Supra/Infra	12	9	12	9	12	9	12	9	84
FF/Intra	21		21		21		21		84
P/NP	42				42				84
Area 3b	84								84

*P, patch; NP, no patch; FF, feedforward; intra, intra-areal; inter, inter-areal; Supra, Supragranular; Infra, infragranular*.

Regularly spaced series (at 130–160 μm except one case with 270 μm) of 50-μm thick tangential sections were cut by vibratome sectioning (see Négyessy et al., 2013; Ashaber et al., 2014). A standard avidin-biotin complex (ABC) protocol (Vectastain Elite ABC kit, Vector Laboratories, Inc.) was used to visualize BDA labeling with nickel-intensified diaminobenzidine (NiDAB) (Sigma-Aldrich) as chromogen (for more details on the procedure, see Négyessy et al., 2000, 2013). First, sections were cryoprotected (30% sucrose in PB) and tissue penetration was enhanced by freezing–thawing. Unbound aldehydes were reduced by borohydride (1% NaBH_4_ in PB, 30 min), and intrinsic peroxidase activity was blocked with 1% H_2_O_2_ in PB (30 min). The sections were then incubated in ABC (1:200 in PB, 0.1M, pH 7.4) overnight at 4°C. After the NiDAB reaction, the sections were osmicated (1% OsO_4_ (Electron Microscopy Sciences) in PB (pH 7.4) containing 5% sucrose for 60 min) and flat embedded in resin (Durcupan ACM, Sigma-Aldrich).

### Data Collection and Analyses

#### Reconstruction of the Axonal Segments

**Figure 1:**
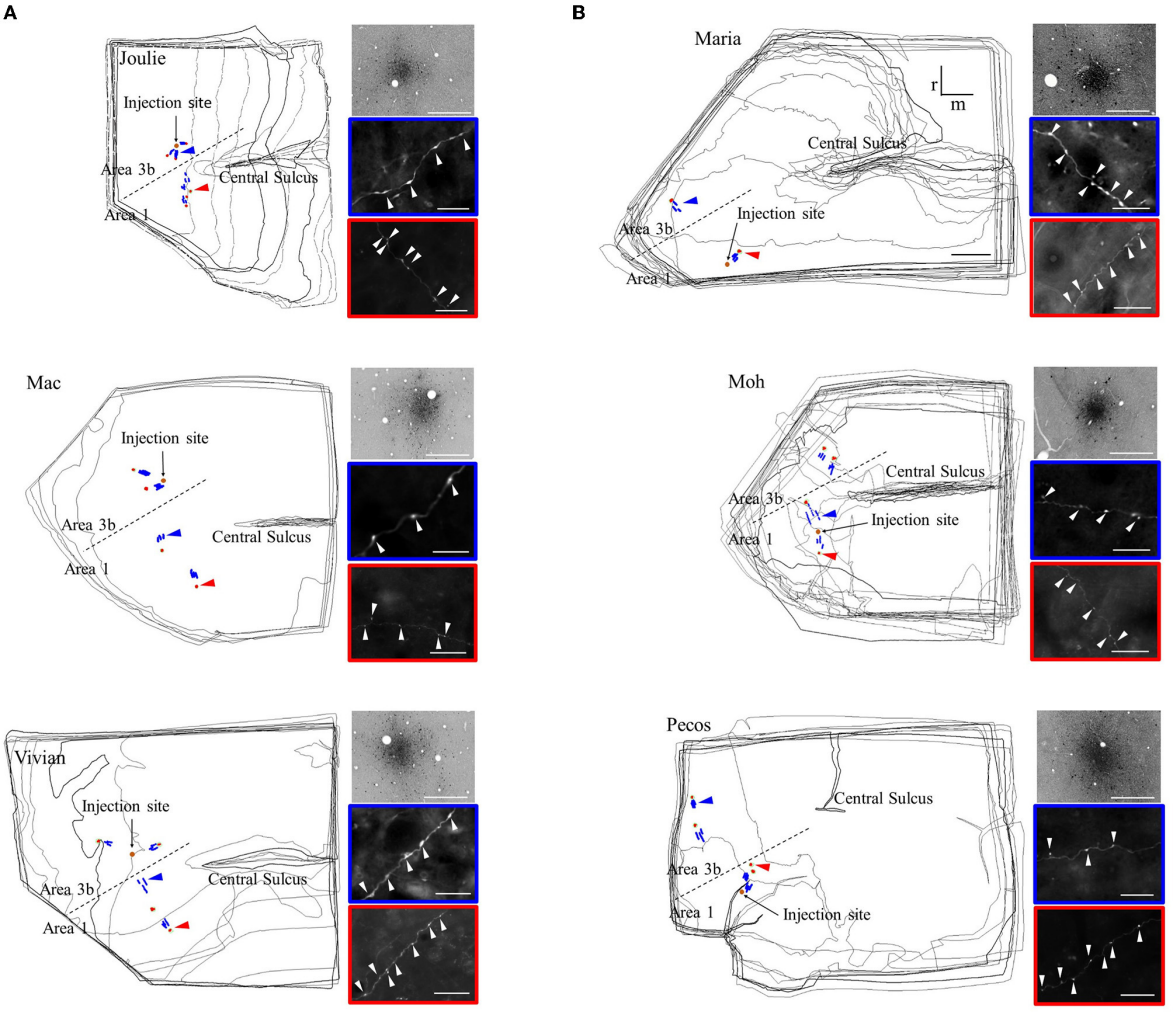
Distribution (left drawings) and light microscopic appearance (right panels) of the reconstructed long-distance axonal segments. **(A)** Area 3b injections and **(B)** area 1 injections. Distribution of the reconstructed axons is shown on the merged series of sections in the six cases. Anterograde labeling is represented by patch-forming axons (red) and no-patch-forming axons (blue) in the hand representation region of areas 3b and 1. The name of cases is indicated in the upper left corner. CS: central sulcus, dashed line: border between areas 3b and 1. Orange circle show the injection site located in the representation of a distal finger pad. The orientation bars on B (case M) apply to all panels; r: rostral, m: medial. Scale bar on B (case M) represents 1 mm, applies also to the other cases. Right panels: light microscopic images enclosed by a color border are taken from the sites indicated by the arrowheads of the same color on the drawings. Top panels show the injection site. Middle and lower panels show, respectively, no-patch axons and patch-forming axonal segments with numerous boutons in the form of varicosities (white arrowheads). Scale bar: 25 μm (right, top), 20 μm (right, middle and bottom).

**Figure 2:**
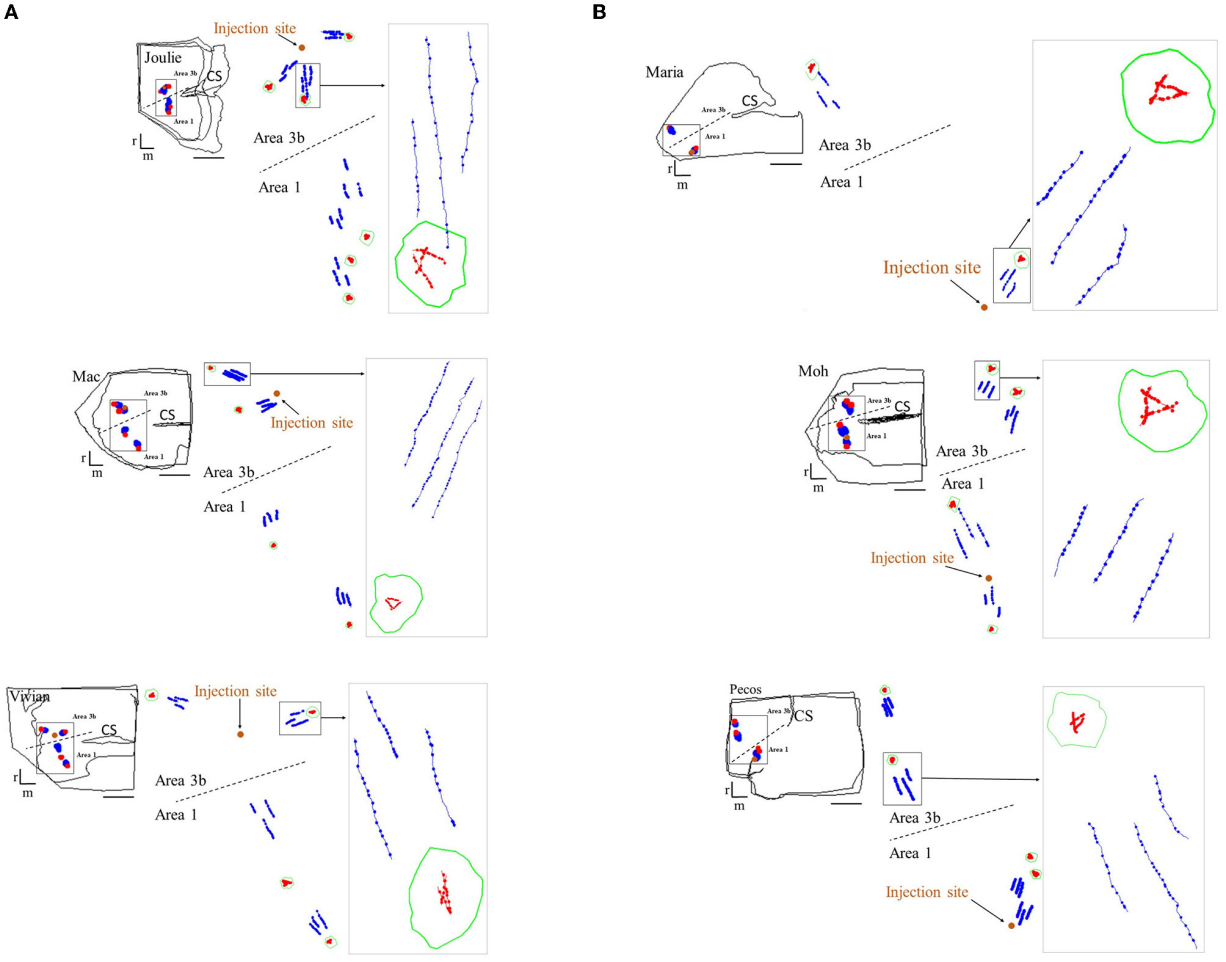
Examples of reconstructions illustrating the morphology of patch and no-patch axonal segments in the six cases. **(A)** Area 3b injections and **(B)** area 1 injections. For each case: top left corner: miniature diagram of the section outlines including the reconstructed axons shown at higher magnification in the middle. Middle: enlarged view of the areas (demarcated by the rectangle in the section outlines on left) including the reconstructed axonal segments. The orange dot shows the location of the injection site. Right: high magnification view of the areas outlined by the rectangle (arrow). Note that, on the right, the high-resolution view is taken from a single section. Axon arborization patches are outlined by green contours. Patch axons and no-patch axons are shown in red and blue, respectively. Boutons formed by the reconstructed axons are marked with dots resulting in the beaded form of axonal segments. Examples include axons from both supragranular and infragranular layers, except for case M, where only supragranular axons can be reconstructed. Note the shorter length, highly variable direction and high bouton density of axons within the patch compared to no-patch axons. Dashed line represents the border between the two areas of the somatosensory cortex. r: rostral, m: medial. Scale bar: 1 mm.

Axonal patches lacking retrogradely labeled somata and spanning the supragranular and infragranular layers (projecting intra-areal and inter-areal in the different cases, see Figure 1) were selected and outlined using Neurolucida (MicroBrightField Europe, E.K.). In each patch, three BDA-labeled axonal segments were reconstructed in three dimensions (3D; Figures 1, 2; Tables 1–**4**). Three labeled axonal segments in each cortical region between the injection site and the patches were also selected and reconstructed. These no-patch axonal segments were directed toward the patches due to the radial spread of axons from the injection sites (Figures 1, 2) (Négyessy et al., 2013; Ashaber et al., 2014). Three-dimensional reconstructions of patch and no-patch axons were made in high resolution (100× objective magnification), allowing faithful reconstruction of the tortuous path of axonal segments and the localization of boutons formed by axons. Most of the boutons were varicosities along axons, only a few terminal-like endings were found (Kisvárday et al., 1997). The two kinds of boutons were not distinguished in the analyses. All reconstructions were made in the gray matter of the injected hemisphere.

#### Measurements and Data Analyses

Quantitative measures of axonal properties were obtained as follows. Axon length, bouton number, and distances between boutons along an axonal segment were retrieved from Neurolucida's Neuroexplorer. The length was measured from the starting point, where the axon appeared at the top of the section. Inter-bouton interval was defined as the axonal distance to the preceding bouton. For axonal thickness measurements, digital images were captured with 100× objective magnification by a two-megapixel CCD camera built into the Neurolucida setup. Thickness measurements were made with ImageJ at three different locations at random along an axon and then averaged for each axon separately.

Bouton density, i.e., the number of boutons in a unit length, was calculated for the full length of an axonal segment. The variability of bouton distribution along an axon was characterized by the standard deviation (SD) of the distances between boutons. Bouton clustering was measured by counting the number of boutons that were farther from each other (in terms of interbouton intervals) than a separation length along an axon as a function of separation length. Separation length was increased from 1 to 150 μm by 1 μm steps. This measurement resulted in a sharp decline in the number of bouton clusters as the separation length increased, reaching a cluster number of one at the full axon length. Conversely, the maximum number of clusters, i.e., the total number of boutons in an axon, would be counted when the separation length became shorter than the distance between any two boutons. To obtain a single value for bouton clustering for each axon, the data were transformed into a log-linear function and clustering was defined by the slope of the fitted line. A larger negative value indicated a larger clustering propensity of boutons along the axon. Supplementary Figure S1A illustrates the measure of bouton clustering after pooling the measures of patch and no-patch axons.

Tortuosity is a measure of the length of an axon relative to the straight line connecting the endpoints. A caveat in determining tortuosity is that the measurements can be sensitive to axon length due to the sharpness of the turns. To handle this issue, it was found that making multiple tortuosity measures along the length of an axon revealed length values that allowed comparisons of tortuosity of different classes of axons (Stepanyants et al., 2004). To determine the length value that best distinguished between patch and no-patch axon segments, we plotted the cumulative change in tortuosity as a function of a fixed 10 μm incremental increase of the axonal length. After separately pooling the measures from patch and no-patch axons, the graphical representation allowed us to identify that the first 40-μm segment of the axons (beginning at the start point) was a length where the tortuosity of patch and no-patch axons clearly differed and exhibited low variability (Supplementary Figure S1B). Consequently, we used as an estimate of the tortuosity of patch and no-patch axons, the tortuosity of the first 40-μm segment.

Directional differences in the projection of axon groups were determined with ImageJ in 2D by ignoring section depth. Each group was formed by selecting three axons found within the supragranular or the infragranular patches, or by selecting three axons outside those patches that these axons were directed towards (see Figures 1, 2). The direction was determined in the 0–180° range relative to a reference line drawn between the injection site and the patches in the individual sections. The ordered projection of individual axons was measured by calculating the difference in the direction of a single fiber's path from its group mean in degrees. A measure of the convergence of boutons (bouton-convergence) was determined in Neuroexplorer. The average distance of a bouton of a reconstructed axon segment from the two nearest BDA-labeled boutons of other axons were measured for each patch and no-patch axon.

For factorial analysis of variance (ANOVA), we averaged the data obtained from each axonal segment localized either in the supragranular or the infragranular layer in the different categories of different cases. The sample size (number of means) contributing to each category is summarized in Supplementary Table S1. Note that due to the lack of infragranular data in case M (see details in Section Number of the Reconstructed Axons and Boutons), laminar comparisons of patch and no-patch axons had to be omitted as data were only available in two cases with area 1 injections.

Statistical analyses were performed in Statistica (version 13., TIBCO Software Inc., 2018, http://tibco.com) and MS Excel. Principal component analysis (PCA) and stepwise logistic regression were done in Statistica. If not mentioned otherwise, measurements and computations were made in MATLAB and Python.

## Results

### Cortical Distribution, Morphology, and Number of the Reconstructed Axons and Boutons

Our sample of anterogradely labeled axons consisted of 144 3D reconstructed axons forming connections within somatosensory areas 3b and 1 as well as between these two areas after focal injection of BDA in areas 3b (*n* = 3) and 1 (*n* = 3) in six squirrel monkeys (Figure 1; Tables 1, 2). An equal number of patch and no-patch axons were selected for analysis; however, a larger number of axonal segments were reconstructed following area 3b (*n* = 84) injections compared to area 1 (*n* = 60) injections (Tables 1, 2). This was mainly due to the low number of axons available in case M, where an oblique cut of the sections prevented the reconstruction of infragranular axons with sufficient length (Tables 1, 2, **4**). Tables 1, 2 also show the number of intra-areal and interareal axons reconstructed in the supragranular and infragranular layers of the different cases. Sample images of the BDA label around each injection site and the spacing of bouton varicosities are shown in Figure 1.

**Table 2:** Total number of reconstructed axons labeled by area 1 injection in the different studied pathways.

**Injected area**	**Area 1**								
	**P**				**NP**				
	**FB**		**Intra**		**FB**		**Intra**		**Total**
**Cases**	**Supra**	**Infra**	**Supra**	**Infra**	**Supra**	**Infra**	**Supra**	**Infra**	
M	3	0	3	0	3	0	3	0	12
Mo	3	3	3	3	3	3	3	3	24
P	3	3	3	3	3	3	3	3	24
Supra/Infra	9	6	9	6	9	6	9	6	60
FB/Intra	15		15		15		15		60
P/NP	30				30				60
Area 1	60								60

*FB, feedback. Other conventions are the same as in Table 1.*

Axons formed numerous boutons both within and outside the axonal patches, irrespective of the injected area, the pathway (intra-areal vs. inter-areal), or the laminar (supragranular vs. infragranular) location (Figure 2; Tables 3, 4). Figure 2 shows the Neurolucida reconstructions of the population of patch (red) and no-patch (blue) axons from each of the six cases. Major morphological differences appeared between patch and no-patch axons in regard to their length (the distance between the start and end points of the reconstructed segments), direction (how parallel they are), and the number of boutons and bouton density. We found that a larger number of boutons were formed by patch axons compared to no-patch axons (Wilcoxon matched pairs test; *p* = 0.001, Tables 3, 4). This observation was even more compelling considering that the length of patch axons was significantly shorter than that of no-patch axons (Wilcoxon matched pairs test; *p* < 0.001, Tables 5, 6). At the level of the supragranular and infragranular layers of the different cases (Supplementary Table S1), the maximum length of patch axons (mean ± SD: 111.42 ± 42.8 μm) was comparable to the minimum length of no-patch axons (mean ± SD: 113.13 ± 43.83 μm) in the sample (Wilcoxon matched pairs test, *p* = 0.59), i.e., the length distributions overlapped. These comparisons suggested that the different length values of patch and no-patch axons collected from the same sections could not be solely a consequence of the plane of sectioning. Details of the number of boutons and the length of axons in relation to the different categorization of axons (patch, no patch, feedforward, feedback, intra-areal or interareal, and area 3b or 1) and cases are summarized in Tables 3–6.

**Table 3:** The number of boutons in different pathways labeled by area 3b injection in different cases.

**Injected area**	**Area 3b**							
	**P**				**NP**				
	**FF**		**Intra**		**FF**		**Intra**		**Total**
**Cases**	**Supra**	**Infra**	**Supra**	**Infra**	**Supra**	**Infra**	**Supra**	**Infra**	
J	63	38	65	38	42	18	42	21	327
Mac	37	34	25	57	20	23	58	55	309
V	42	62	20	27	38	26	34	24	273
Supra/Infra	142	134	110	122	100	67	134	100	909
FF/Intra	276	232	167	234	909
P/NP	508				401				909
Area 3b	909								909

*Conventions are the same as in prior tables*.

**Table 4:** The number of boutons in different pathways labeled by area 1 injection in different cases.

**Injected area**	**Area 1**							
	**P**				**NP**			
	**FB**		**Intra**		**FB**		**Intra**		**Total**
**Cases**	**Supra**	**Infra**	**Supra**	**Infra**	**Supra**	**Infra**	**Supra**	**Infra**	
M	54	0	46	0	31	0	31	0	162
Mo	52	43	21	50	28	36	40	29	299
P	37	43	28	21	41	50	44	25	289
Supra/Infra	143	86	95	71	100	86	115	54	750
FB/Intra	229	166	186	169	750
P/NP	395				355				750
Area 1	750								750

*Conventions are the same as in prior tables*.

**Table 5:** The length [mean ± standard deviation (SD)] of the reconstructed axonal segments of the different pathways labeled by area 3b injection.

**Injected area**	**Area 3b**				
	**P**		**NP**		**Grand avg ± D**
**Cases**	**FF**	**Intra**	**FF**	**Intra**	
J	59.96 ± 12.17	61.21 ± 10.64	123.13 ± 40.86	105.67 ± 31.21	87.49 ± 11.57
Mac	62.25 ± 17.09	108.85 ± 30.39	195.8 ± 64.81	224.63 ± 137.55	147.88 ± 46.71
V	107.6 ± 55.43	85.3 ± 15.64	183.4 ± 41.21	184.88 ± 53.87	140.29 ± 15.93
FF/Intra	76.60 ± 19.33	167.44 ± 8.38	171.73 ± 11.21	125.22 ± 45.42	135.24 ± 14.61
P/NP	122.02 ± 5.47		148.47 ± 17.10		135.24 ± 5.81
Area 3b	135.24 ± 5.81				135.24 ± 5.81

*Conventions are the same as in prior tables*.

**Table 6:** The length (mean ± SD) of the reconstructed axonal segments of the different pathways labeled by area 1 injection.

**Injected area**	**Area 1**				
	**P**		**NP**		**Grand avg ± sd**
**Cases**	**FB**	**Intra**	**FB**	**Intra**	
M	86.06 ± 10.85	70.73 ± 3.21	139.96 ± 19.00	100 ± 25.28	99.19 ± 8.32
Mo	102.45 ± 31.21	114.83 ± 27.30	179.9 ± 35.82	337.98 ± 158.98	183.79 ± 55.30
P	64 ± 11.59	63.76 ± 12.65	291.76 ± 126.65	179.61 ± 52.59	149.78 ± 46.84
FF/FB	84.17 ± 9.42	83.11 ± 9.91	203.87 ± 47.37	205.86 ± 57.67	144.25 ± 20.44
P/NP	83.64 ± 0.24		204.87 ± 5.15		144.25 ± 2.45
Area 1	144.25 ± 2.45				144.25 ± 2.45

*Conventions are the same as in prior tables*.

### Distinctively Different Axonal Segments Within and Outside Patches

**Figure 3:**
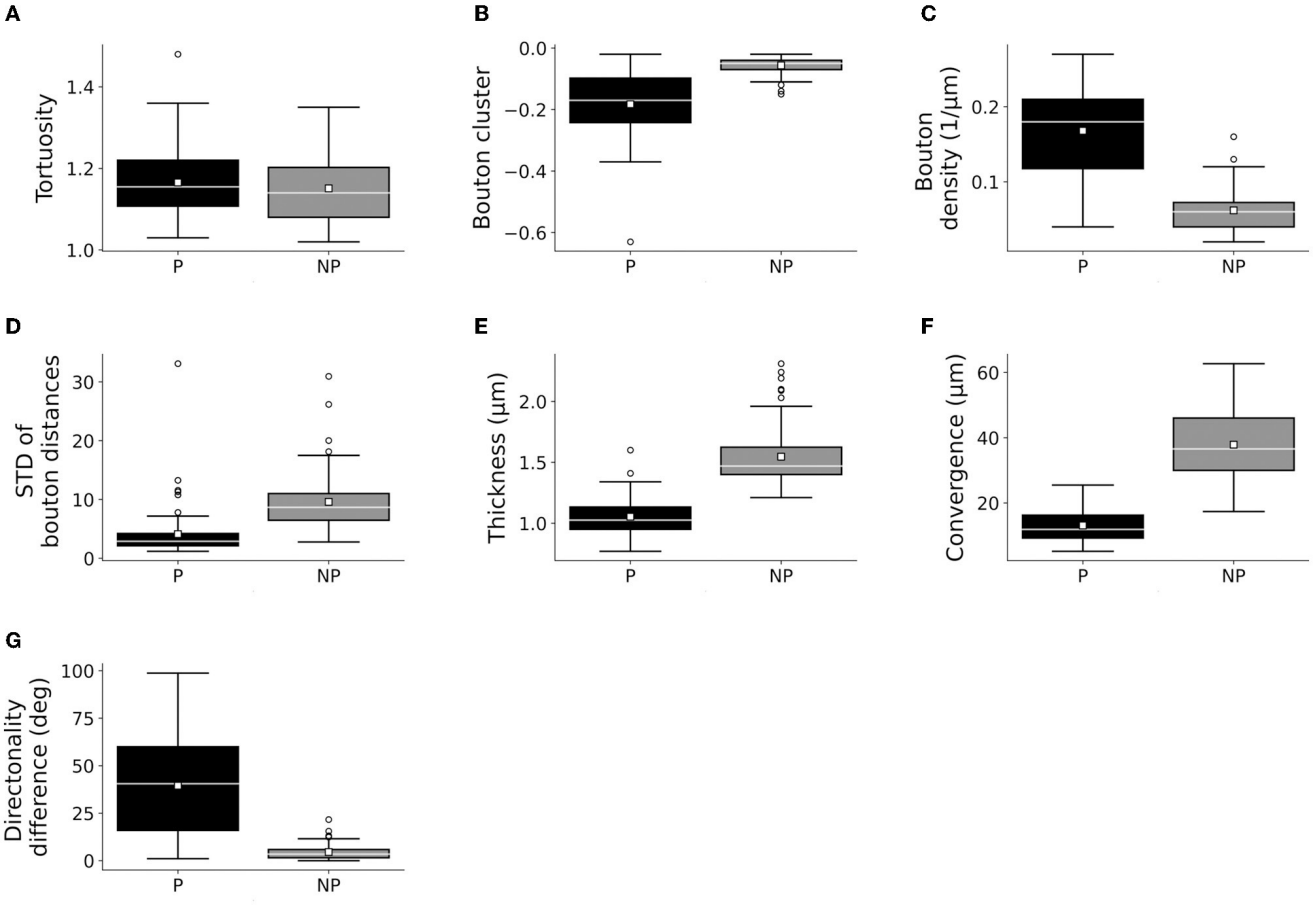
Box plots of measured bouton and axonal characteristics of patch (P: 72) and no-patch (NP: 72) axons. **(A)** Tortuosity (the first 40 μm segment of the axons beginning with the starting point), **(B)** bouton clustering (lower values indicate higher clustering), **(C)** bouton density, **(D)** bouton distance SD (SD of interbouton intervals along axons), **(E)** thickness, **(F)** convergence (or bouton-convergence, the distance of a bouton of a reconstructed axon from the two nearest boutons of unconnected axons), and **(G)** difference of the directionality of the axonal segments. Here and in the following plots, panels **(A,E)** show individual axonal properties and **(F,G)** presents collective axonal properties. Horizontal line: median, small square: mean, box: 25–75%, whiskers: non-outlier range, Circles: outliers representing data > 1.5 × height of the box.

**Figure 4:**
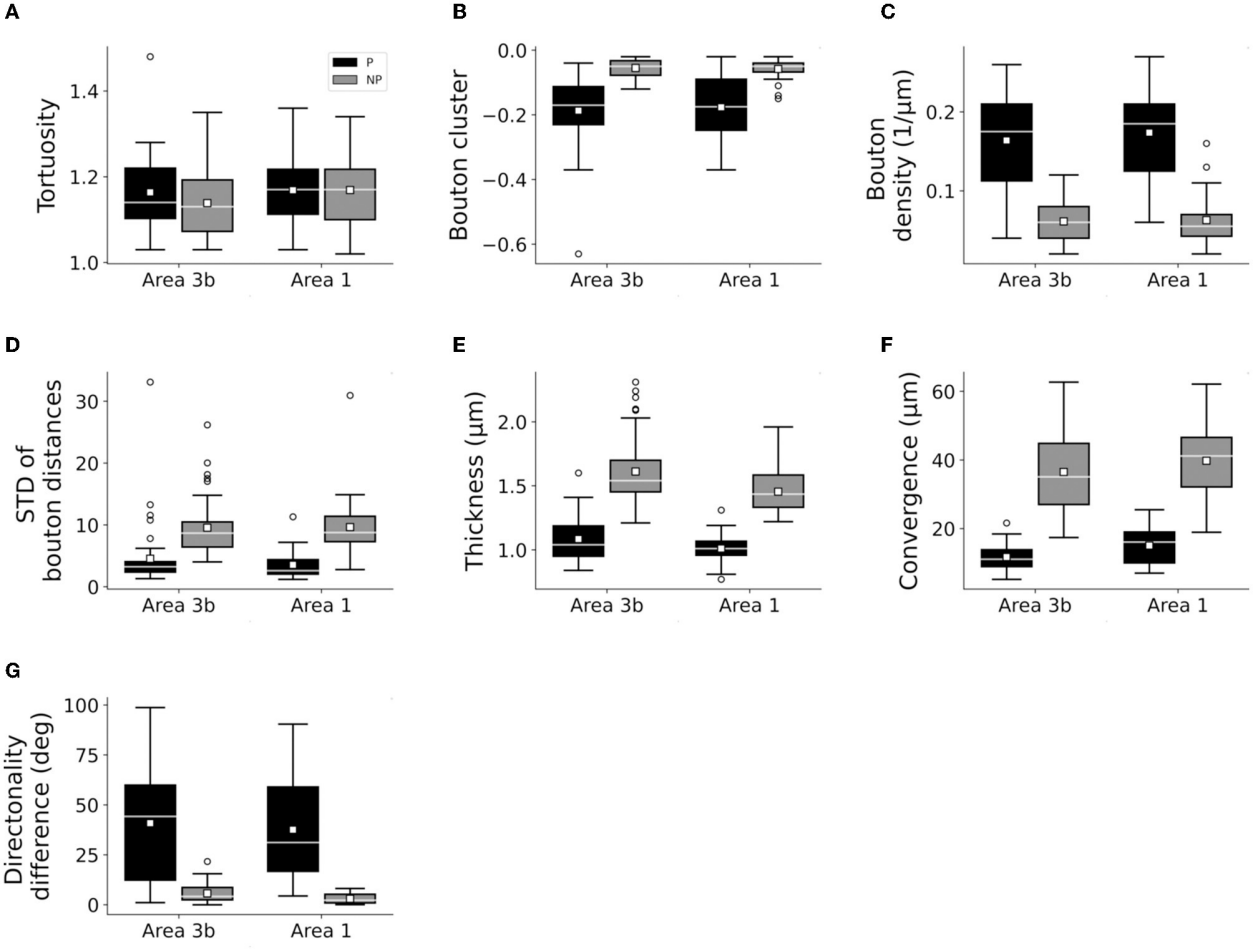
Box plots showing similar characteristics of patch (P) and no-patch (NP) axons following the injection of areas 3b and 1. Area 3b: *n* = 42 P, 42 NP axons. Area 1: 30 P, and 30 NP axons **(A–G)**. Conventions are the same as in Figure 3.

Only the classification of axons by patch designation (patch or no patch) showed differences in the distribution of the quantitative properties of axons (Figures 3, 4; Supplementary Figures S2–S5). Greater bouton density, convergence, clustering, and more consistent bouton spacing were observed in patch than in no-patch segments. Notably, SD as a measure of consistency of bouton spacing was scaled to the mean and the inter-bouton interval exhibited a similar coefficient of variation (CV = mean/SD) in the case of patch and no-patch axons (mean ± SD, patch: 0.57 ± 0.07; no patch: 0.54 ± 0.03; Wilcoxon matched pairs test, *p* = 0.45). Axon measures showed thicker axons in no-patch segments and more similar directionality of axons than in patch segments with no differences in tortuosity (Figure 3). These morphological distinctions were independent of whether the projections originated in area 1 or 3b (Figure 4) or whether axons were intra-areal or interareal or feedback (projection from areas 1 to 3b) or feedforward (projection from areas 3b to 1) projections or whether the segments were located in supragranular or infragranular layers (Supplementary Figures S2–S5). Factorial ANOVA supported these observations. After including the categorical variable injection area (areas 3b, 1), patch designation, and pathway type (intra-areal and inter-areal), factorial analysis resulted in a significant major effect only between patch and no-patch axons (Table 7). Interactions were not significant. *Post hoc* comparisons resulted in significant differences in all variables except the measure of tortuosity (Table 7).

**Table 7:** The results of multivariate test of significance by factorial analysis of variance (ANOVA; df: 7, 30) and *post hoc* comparisons (Scheffe test, df: 36).

**ANOVA**	**F**	***p* values**	**Scheffe**	***p* values**
Injection-Area (IA)	1.09	0.395	Tortuosity	0.672186
Patch Designation (PD)	72.61	0.000	Bouton cluster	2.35E-09
Areal Localization (AL)	0.94	0.490	Bouton density	6.93E-12
IA ^*^ PD	0.39	0.899	Bouton distance SD	6.93E-07
IA ^*^ AL	0.49	0.837	Bouton-convergence	1.03E-14
PD ^*^ AL	1.41	0.238	Directionality difference	6.88E-14
IA ^*^ PD ^*^ AL	0.49	0.831	Thickness	8.20E-13

Significant p-value is highlighted by red. Injection-Area: area 3b vs. area 1; patch designation: P vs. NP axons; areal localization: intra-areal vs. inter-areal axons. Interactions are indicated by the “*” symbol. Other conventions are the same as in prior tables.

Based on these observations, we determined the relative contribution of the different variables to the patch-like properties of axons. To do this, we utilized PCA to account for variability, followed by stepwise logistic regression to determine the predictors of patch designation across the different variables.

### Structural Properties Distinguishing Patch and No-Patch Axons

#### Principal Component Analysis

**Figure 5:**
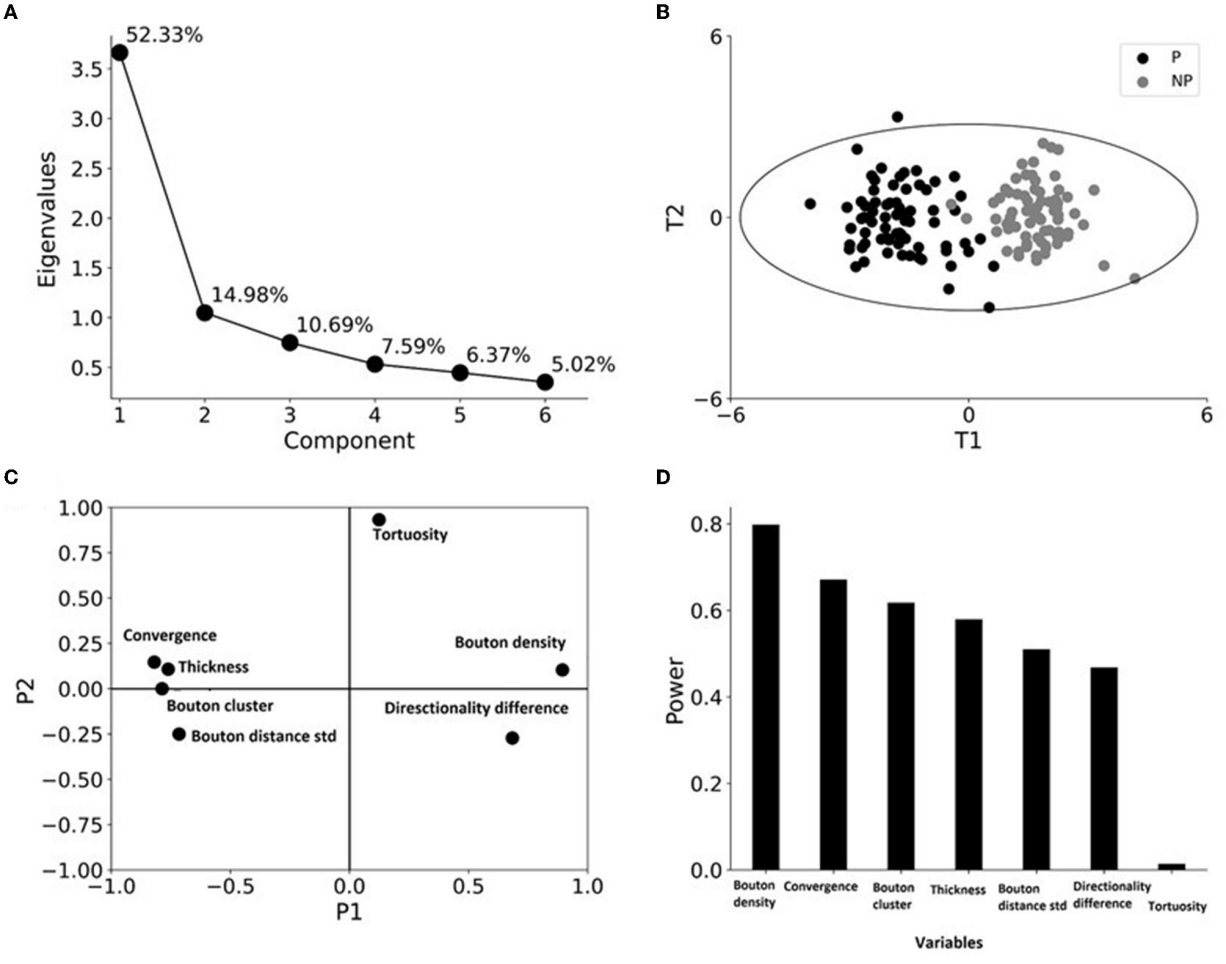
Principal component analysis (PCA) identifies clustering only by patch designation. **(A)** A scree plot showing the eigenvalues and the percentage of variance explained by the six principal components. **(B)** Case-wise analysis shows the tendency of the grouping of patch (P) and no-patch (NP) axons, black and gray circles, respectively, along the two principal components. Distribution of scores (distances of the transformed values of the variables from the origin along the PCs) of axons for the first principal component (T1) plotted against the scores for the second principal component (T2). The ellipse outlines ± 3 SD and indicates the presence of a single patch outlier in the data set. **(C)** Distribution of loading factors (transformed values showing the contribution of the variable to the PCA model) P1 and P2 for the first and second principal components. The greater a variable is away from zero, the more influence that variable has. A diagonal position in opposite quadrants means a negative correlation between the variables. **(D)** The importance of the variables is measured by the modeling power, which is defined as the SD explained.

We performed PCA to see the combined effect of the variables as well as their power in distinguishing patch and no-patch segments. Before the analysis, data were normalized such that each variable had a zero mean and unit SD. The PCA resulted in two significant PCs (Figure 5A). The two PCs differed as PC1 explained 52% of the variance in contrast to the 15% explained by PC2. More importantly, in agreement with the results of ANOVA (Table 7), PCA resulted in clustering of axons according to their patch designation (Figure 5B). In addition, patch and no-patch axons were grouped by PC1 without additional separation by adding PC2. This observation was in accordance with the different magnitudes of variance explained by the two PCs (Figure 5A). Figure 5C shows that all the variables, which exhibited a significant difference between patch and no-patch axons by ANOVA (Table 7), contributed largely to PC1 with only small loadings on PC2. In contrast, tortuosity had a high loading on PC2 and minimally contributed to PC1. Analysis of the importance of the variables by their explained variance in PC1 showed that bouton density followed by bouton-convergence had the highest power while tortuosity exhibited almost no importance (Figure 5D).

#### Stepwise Logistic Regression

To identify the variables that best determine the patch designation of axonal segments, we applied logistic regression. First, we evaluated marginal effects to select variables with a significant outcome in distinguishing patch and no-patch axons (Table 8). All variables that exhibited a significant difference by ANOVA (Table 7) showed a significant contribution to the regression. Significant variables were then entered into a stepwise model according to the model fit statistics (estimation of the constant by minimizing the fitting error) in the marginal model (Table 9). Bouton-convergence provided the best fit in the regression model and was first entered into the model, which resulted in a significant improvement in classification (Table 10). In the next step, ignoring bouton-convergence and using the remaining six variables, only bouton density exhibited a significant contribution to a marginal model (Supplementary Table S2). This observation was consistent with the PCA result (Figure 5D). Entering bouton density into the stepwise model with bouton-convergence had the highest predictive power of patch designation of axonal segments (Table 10). Subsequent marginal models showed no significant contribution of any of the remaining five variables (i.e., without bouton-convergence and bouton density) to the regression (Supplementary Table S3). The estimated constants in the two-step model are summarized in Table 11. The sign of the constants shows that bouton-convergence, i.e., the distance of boutons formed by neighboring, unconnected axonal segments, was positively correlated (indicating larger distances) with the probability of being a no-patch axon whereas bouton density showed negative correlation with this probability.

**Table 8:** The results of marginal model with all seven variables (codes: *P* = 0, NP = 1).

**Effect**	**Somers' D**	**Constant**	**Pr > Chi2**	**df**
Tortuosity	0.097	−2.139	0.292	1
Bouton cluster	0.847	43.056	5.23E-08	1
Bouton density	0.870	−47.913	1.17E-08	1
Bouton distance SD	0.794	0.399	1.05E-08	1
Bouton-convergence	0.982	0.603	7.73E-05	1
Thickness	0.947	16.417	3.97E-08	1
Directionality difference	0.890	−0.256	2.01E-06	1

*The values showing significant fit are highlighted in red*.

**Table 9:** Model fit statistics for the seven variables.

**Marginal predictors**	**AIC**	**AICC**	**BIC**	**R2**
Tortuosity	202.502	202.587	208.442	0.010
Bouton cluster	101.874	101.959	107.814	0.676
Bouton density	91.959	92.044	97.898	0.719
Bouton distance SD	146.747	146.832	152.686	0.435
Bouton-convergence	37.852	37.937	43.791	0.912
Thickness	62.749	62.834	68.688	0.832
Directionality difference	87.049	87.134	92.988	0.740

*AIC, Akaike information criterion; AICC, AIC corrected for small sample size; BIC, Bayesian information criterion; R^2^, adjusted coefficient of determination*.

**Table 10:** Summary of building the stepwise logistic regression model.

**Step no**.	**Model variables**	**df**	**Wald**	**Wald *p*-value**	**Somers' D**	**KS statistic**	**KS *p*-value**
1	Bouton-convergence	1	15.622	7.733E-05	0.982	0.903	6.552E-26
2	Bouton-convergence	1	9.927	0.002	0.989	0.917	1.062E-26
	Bouton density	1	4.308	0.038			

*Significant values are highlighted in red*.

**Table 11:** Parameter estimates (modeled probability that *P*/NP = NP) in the final model including variables that significantly improved the model fit.

**Effect**	**Constant**	**Standard error (SE)**	**Wald Stat**.	**Lower CL 95.0%**	**Upper CL 95.0%**	***p***
Intercept	−7.993	3.513	5.177	−14.879	−1.108	0.023
Bouton-convergence	0.497	0.158	9.927	0.188	0.806	0.002
Bouton density	−26.612	12.821	4.308	−51.741	−1.482	0.038

*The p-values of bouton-convergence and bouton density correspond to the Wald p-value in Table 10*.

**Figure 6:**
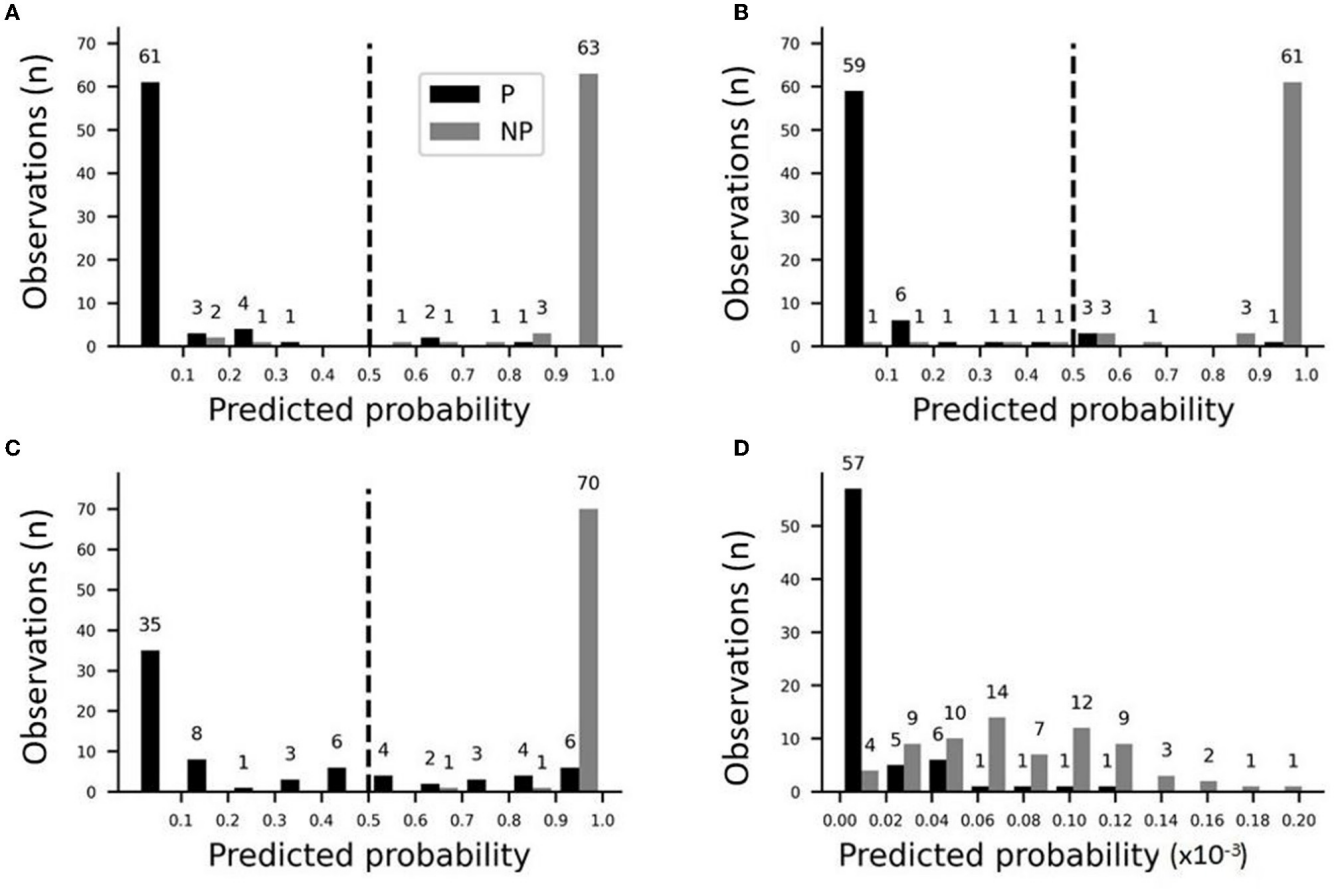
Predicted probabilities of identifying axon types (P vs. NP) in stepwise logistic regression models by applying a threshold of 0.5. Distribution of predicted probabilities: **(A)** The final model with bouton-convergence and bouton density as the predictor variables; **(B)** A model with bouton-convergence as a single predictor variable; **(C)** Contribution of bouton-convergence in the final model; **(D)** Contribution of bouton density in the final model.

**Figure 7:**
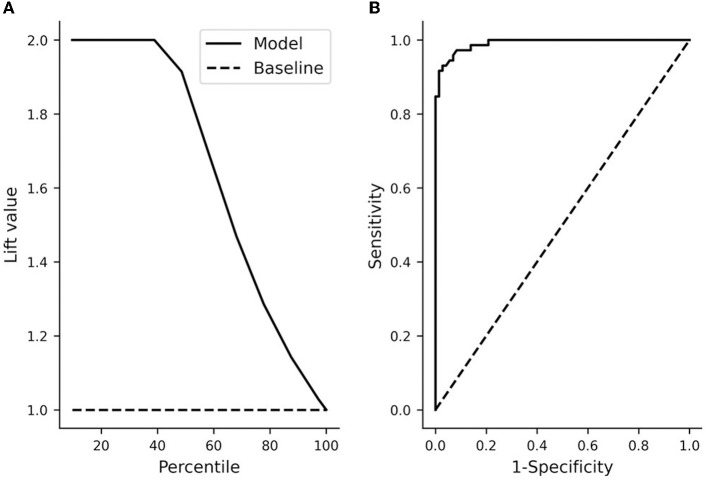
Overall model performance of the stepwise logistic regression. **(A)** The lift chart shows the ratio (lift value) of correct predictions based on deciles (percentile) of the sample (i.e., axons) ordered by decreasing the predicted probability to that of a random sample. At the full sample size (100%), the lift value equals 1. In the final model (solid line) with a threshold value of 0.5 for up to about 40% of the axons, there were two times as many correct predictions as in the case of the same percentage of axons selected randomly (dashed line). Also, a quick decline in the elevation value after about 40% shows that the inclusion of more axons did not increase the ratio of correct prediction relative to that of a random sample. **(B)** Receiving operating characteristics (ROC) of the model. The area under the ROC curve (0.99) indicates a very high accuracy. The solid line shows the accuracy of the model whereas the dashed line shows the performance if axons were randomly classified. Sensitivity: true positive rate, 1-specificity: false positive rate. A perfect classifier would have a true positive rate of 1 with a false positive rate of 0.

The performance of the model was checked by plotting the distribution of predicted probabilities from stepwise logistic regression (Figure 6). The full model that included bouton-convergence and bouton density resulted in only a few erroneous classifications (Figure 6A). In the full sample, only three no-patch axons (gray bars to the left of the dotted line) were categorized as patch axons; similarly, only three patch axons were misidentified as no-patch axonal segments. As there were so few misidentifications, the differences between the observed and predicted rates of being in one of the selected categories (no patch in our case) were only slightly different in the full model (Supplementary Figure S6). Accordingly, the difference between the observed and predicted rates was distributed around zero (Supplementary Figures S6A,B) and the frequencies of occurrence and means of these two rates were nearly identical (Supplementary Figures S6C,D). Interestingly, a closer look at the distribution of predicted probabilities showed that bouton-convergence alone was an excellent predictor of patch designation (Figure 6B). In the final model, bouton-convergence outperformed bouton density in detecting patch axons, whereas the opposite was true in recognizing no-patch axons (Figures 6C,D). Overall, bouton-convergence performed better than bouton density in predicting patch designation by our stepwise logistic regression model. In spite of this, the model showed high prediction power and accuracy (Figure 7). As shown in Figure 7A, selecting as few as 10% of the axons that were best predicted by the model doubled the ratio of correct predictions to that found in a randomly selected 10%. This advantage of the model against random sampling disappeared only when using larger than 40% of the axons. Also, an accuracy of 0.99 was identified by the receiving operating characteristic (ROC) curve (Figure 7B). The sharp rise of the curve indicates that the model (solid line) had a very high true positive rate with a very low level of false positive classification rate. Our model achieved a maximum sensitivity (true positive rate) with a low false positive rate of approximately 0.2 (1−Specificity). Optimal performance resulted in 0.96 (138/144) true positive and 0.04 (6/144) false positive rates (Figure 6A).

## Discussion

### Overview of the Findings

In this study, we explored individual (tortuosity, thickness, bouton density, variability of inter-bouton distance, and clustering tendency of boutons) and collective (distance of the closest boutons of unconnected axonal segments and directional difference of the axonal segments) morphological variables of bouton-forming axonal segments that are relevant to signal transmission and best predict the properties of a patch. We found that, except for tortuosity, all other variables examined differed significantly between axons with varicosities within patches and outside patches. Most notably, we identified proximity of boutons of unconnected axonal segments (bouton-convergence) as the variable with the strongest predicting power of patch designation. In addition to bouton-convergence, bouton density also contributes significantly to the predictive power of an axon's patch designation. As patches are characteristic of mesoscale connectivity in the cerebral cortex of carnivores and primates, an important finding is that in the different pathways (intrinsic, feedforward, or feedback) and layers (supragranular and infragranular) studied axons do not vary in their properties; axons are differentiated exclusively by their patch designation. Taken together, patch and no-patch domains of horizontal, long-distance axons exhibit distinctive morphological properties, which suggest their complementary role in cortical computation and support the modular organization of cortical interactions.

### Limitations

One of the methodological limitations is that sampling of the serial sections used in this study does not allow tracing back to the origin of the axon. Therefore, it is possible that separately reconstructed axonal segments belong to the same neuron. While this possibility cannot be ruled out, single-cell reconstructions show that recurrent horizontal axonal branches of cortical spiny neurons are oriented in different rostro-caudal and medio-lateral directions, instead of raising multiple parallel collaterals (Buzás et al., 2006; Binzegger et al., 2007). Based on this observation, we expect our sample to include at most a negligible number of linear no-patch axonal segments, which belong to the same neuron. Within patches, the chance that the sampled segments belonging to the same axonal arborization may be higher. However, the distal branches of fully reconstructed neurons typically branch in a Y-shape and are usually divergent, unlike our sample, in which patch axons are overlapping (Buzás et al., 2006; Binzegger et al., 2007). Studies show a strong laminar preference of horizontal connections of neurons in different layers of the visual cortex (Kisvárday, 2016; Martin et al., 2017). In this study, with a single exception, we reconstructed patch and no-patch axons from more than one section with wide inter-section gaps (i.e., in widely separated layers), and in different horizontal locations, which also decreases the possibility of sampling axons from the same neuron.

Our approach does not allow the unequivocal identification of the type of parent neuron of the axonal segments studied. Although, as described in section Introduction, long-distance horizontal connections are known to be mostly formed by pyramidal neurons, there are transcolumnar and projective GABAergic neurons that can be anterogradely labeled in our experiments (DeFelipe et al., 2013). Even if this is the case, the number of labeled GABAergic axonal segments probably forms a small fraction of the total number of axonal segments studied. The population of GABAergic neurons with a long-distance projection is a subfraction of the total number of GABAergic cells, which form 10–30% of the neurons in the cerebral cortex (DeFelipe et al., 2013). Furthermore, qualitative and quantitative morphological observations are in agreement with the horizontal distribution of axons of individual pyramidal cells. As shown previously, the axonal boutons of pyramidal neurons mostly appear in the form of varicosities similar to the one found here (Kisvárday et al., 1997). Furthermore, in cat visual cortex, single-cell labeling revealed that intrinsic horizontal connections of pyramidal neurons consist of linear segments that terminate in a rich arborization of short, distal branches (Buzás et al., 2006; Muir and Douglas, 2011). Also in cat visual cortex, the horizontal pattern of boutons formed by nearby pyramidal neurons is consistent with the distribution of patch and no-patch domains described here (Kisvárday et al., 1997; Buzás et al., 2006). Together, these observations suggest that our sample consisted of axons originating from pyramidal neurons. Specifically, no-patch axons probably represent the linear segments whereas patch axons can correspond to the terminal arborizations of the horizontal axonal connections.

The chance of including labeled axons by retrograde backfilling of neurons projecting to the injection site has been discussed in our previous studies (Négyessy et al., 2013). Our observations about the apparent lack of thalamocortical labeling and secondary labeling of patches formed by neurons after probable backfilling suggest that these factors did not significantly influence our results. Horizontal sectioning may also have resulted in inconsistencies in the laminar localization of the axonal segments, as discussed earlier (Pálfi et al., 2018). In that study, we found a laminar preference of connectivity similar to that as shown before (Burton and Fabri, 1995). In our samples, the majority of patches and boutons were localized in the supragranular layer (Pálfi et al., 2018), which is consistent with the prevalence of patchy organization of horizontal connections in layers 2/3 (Douglas and Martin, 2004; Kisvárday, 2016).

It should also be noted that our thickness measurements of the axons probably indicate larger values than in reality due to the relatively low resolution of the digital camera attached to the Neurolucida setup and also to the relatively larger scattering of light through the osmicated and resin-embedded sections. Therefore, these values are not comparable to those published in the literature (Anderson et al., 2002; Innocenti and Caminiti, 2017; Koestinger et al., 2017). However, this systematic effect of measurement would not affect the results of the comparisons made in this study.

### Convergence and Divergence of Long-Distance Horizontal Connections

Our analyses indicate that the tortuosity of bouton-forming axonal segments of pyramidal cells is an invariable property and, therefore, neurons save material and use other strategies in finding their specific targets (Anderson et al., 2002; Budd et al., 2010). We found that all variables related to bouton placement differed significantly between patch and no-patch axons. It should be noted that the CV of inter-bouton intervals observed here is in agreement with the 0.5 reported by Shepherd et al. (2002) and indicates that bouton spacing is neither completely random (CV = 1) nor regular (CV = 0). Our findings support that the subrandom distribution of interbouton intervals is a feature of unmyelinated axonal branches in the brain (Hellwig et al., 1994; Shepherd et al., 2002). Even though bouton spacing variability is a constant fraction of the mean spacing along axons, irrespective of the type of neuron or branch order, only boutons on the terminal branches of lateral connections form clusters (Anderson et al., 2002; Shepherd et al., 2002; Binzegger et al., 2007). These observations are supported by our results showing larger bouton densities, clustering tendency, and smaller variability of interbouton intervals along the axonal segments within patches compared to no-patch segments. Furthermore, boutons of unconnected axonal segments are significantly closer to each other within patches than outside of it. These findings are in line with observations showing the overlapping distribution of terminal axon arborizations of a population of nearby pyramidal neurons in cat visual cortex (Kisvárday et al., 1997; Buzás et al., 2006). By showing higher bouton densities within patches compared to no-patch domains, we confirmed our previous observations in primate somatosensory cortex (Négyessy et al., 2013; Ashaber et al., 2014). In these previous studies, we showed that bouton density is significantly higher in axonal patches than in any other locations including regions with BDA-labeled tracks of long-range horizontal axons.

In addition to bouton spacing, no-patch axons were significantly thicker than patch axons, which is in accordance with axon branching order, given that patches are formed by the most distal branches (Buzás et al., 2006; Binzegger et al., 2007). The direction of axonal segments also differs between patch and no-patch domains. The highly varying path of an axon within patches can promote convergence in contrast to the nearly parallel direction of no-patch axons, especially considering that axons were reconstructed in ~50-μm thick sections. Viewing from a larger scale, no-patch axons extend radially from an injection site similar to the distribution of patches around the injection site (Négyessy et al., 2013; Ashaber et al., 2014; Pálfi et al., 2018).

Our application of multivariate data analysis techniques further revealed what makes patch and no-patch domains fundamentally different cortical sites. PCA resulted in a clear clustering of patch and no-patch axons based solely on the first PC, which explained more than half of the variance in the data. Although, with the exception of tortuosity, all variables exhibited a relatively high load on the first PC, the analysis showed that bouton density and bouton-convergence have the largest distinguishing power. To see which of these factors has the highest predicting power of patch designation, we applied stepwise logistic regression. The model identified bouton-convergence with the highest predicting power. Bouton density also contributed significantly to the model fit; however, this parameter had far less predicting power, as shown by the observed probabilities. Altogether, these findings indicate that patches are special cortical loci that can attract axons of a population of nearby pyramidal neurons, which results in the convergence of bouton clusters formed by the distal terminal arborizations (Buzás et al., 2006; Binzegger et al., 2007). Similar evidence for bouton distributions obtained by labeling a small population of neurons and pooling single neuron reconstructions also suggest a highly overlapping distribution of the final terminal arborizations in the cat visual cortex (Kisvárday et al., 1997; Buzás et al., 2006; Martin et al., 2014, 2017).

One of the major principles governing patch formation is probably selectivity for different molecular cues. Reaction-diffusion models can explain patch formation (Bauer et al., 2014). However, pyramidal neurons exhibit a large variability on the basis of molecular composition (Lodato and Arlotta, 2015; Zeng and Sanes, 2017). Considering the multitude of component molecules, some degree of neuronal similarity can be dispersed throughout the cortical mantle and result in the diversity of target sites found in the case of no-patch axons. In such a scenario, the strength of molecular similarity can determine the specificity of target sites of a population of cortical neurons. Another probable factor determining the target neurons is the similarity of functional properties with the presynaptic neuron (Kaas, 2012). Similar rules seem to govern the formation of long-distance interareal, feedforward, and feedback connections. The understanding of how chemical and activity dependent mechanisms interact in the formation of the different cortical circuits requires further investigation.

### Functional Considerations

In the sensory cortex, feedforward interarea input is responsible for activation hotspots or “imprint” (Chavane et al., 2011) at the population level and the formation of the classical receptive field (RF) of neurons (somatosensory cortex: Favorov and Whitsel, 1988; Chen et al., 2003; Friedman et al., 2008; and the visual cortex: Kisvárday et al., 1997; Angelucci et al., 2017). On the other hand, lateral intra-areal connections are thought to transmit contextual information and are responsible for the configuration of extra-classical RF together with feedback interarea connections (Kisvárday, 2016; Angelucci et al., 2017; Chavane et al., 2022). However, how synaptic computations bring the function of these pathways into effect is not completely understood (Douglas and Martin, 2007; Boucsein et al., 2011; Harris and Mrsic-Flogel, 2013; Muller et al., 2018; Rockland, 2018; Chavane et al., 2022). In this study, we made a distinction between the efferents of a population of nearby projection neurons, which is independent of whether the projection pathway is intra-areal, feedforward, or feedback. We found a major difference between patch and no-patch axonal segments. Patches are identified as convergence spots of efferents formed by a population of neighboring neurons, in contrast to the rather parallel spread of neighboring no-patch axons. Notably, this distinction applies to all connections studied, specifically intra-areal, feedforward, and feedback. Also, patch and no-patch characteristics of the axons are independent of the laminar localization. These observations suggest a fundamental similarity in the computation performed by the different pathways. In contrast to the structural properties of the feedforward thalamocortical synaptic boutons, which support their driving functions (Negyessy and Goldman-Rakic, 2005; Petrof and Sherman, 2013), the ultrastructural characteristics of the cortico-cortical synaptic boutons are more homogenous (Ashaber et al., 2020). These electron microscopy findings provide further hints to the importance of convergence in cortical computation.

The convergence of afferents is recognized as the substrate for increasing the effectiveness of signal transmission through the neuronal network. This recognition is supported by the observation that cortical microstimulation can induce a patchy pattern of activation (Roe et al., 2015; Xu et al., 2019; Friedman et al., 2020). Patches can provide the functional bias found in the case of orientation tuning in the visual cortex (Kisvárday, 2016). In contrast, no-patch axons can diversify the input via conveying contextual information to postsynaptic neurons (Kisvárday, 2016; Chavane et al., 2022). Consequently, neurons with overlapping RFs can significantly shape the response specificity of sensory cortical patches. The functional similarity of the neurons forming the patchy projection argues against the lack of specificity of sensory cortical interactions (Chavane et al., 2022). Techniques sensitive to subthreshold changes in membrane potential can reveal input diversity provided by non-convergent projections of no-patch axons (Chavane et al., 2022). The recognition that no-patch regions of a population of neurons probably interact with different functional neuronal populations can explain the formation of cortical circuits that can support both functional specificity and variability. This observation is consistent with the role of patch domains in the emergence of signal correlations of the neuronal responses whereas the no-patch domain can contribute to noise correlations (Panzeri et al., 2015; Bányai and Orbán, 2019).

Neural activity can spread at different velocities inside and outside the patches due to the different axon thicknesses. However, given the short length of the distal branches, it is reasonable to assume that larger bouton densities compensate for the slower conduction speed within the patches. Such compensation in conduction velocity would critically depend on the relative diameter sizes between the axon and the bouton, where a larger difference results in a longer lag in signal propagation (Segev and Schneidman, 1999; Alcami and El Hady, 2019). In addition, a bouton density higher than a critical value can result in a nonlinear increase in conduction delay (Segev and Schneidman, 1999). Further uncertainties in conduction speed can be introduced by activity-dependent changes in axon thickness and bouton size (Alcami and El Hady, 2019). How these factors affect the spread of activity along patch axons remains to be tested. Until these issues are clarified, as a working hypothesis, one can assume a similar dynamic in the two bouton-forming axonal domains of the cerebral cortex.

In our previous studies, we explored the direction of information flow within the circuitry of areas 3b and 1 (Wang et al., 2013; Pálfi et al., 2018). The picture that emerged from that data is consistent with the findings of Polack and Contreras (2012) who found that in the mouse visual cortex information is rapidly forwarded to numerous cortical areas, simultaneously triggering slower serial processing within areas, which can be influenced by the later feedback component in population activity (Semedo et al., 2022). Patchy organization is a characteristic feature of the wiring motif of the mesoscale network of the cerebral cortex of higher order mammals (Roe, 2019). Patches provide distributed modules of axonal convergence that are well positioned to function as sites for the synchronization of neural activity proceeding at rapid and slower time scales. In contrast, due to horizontal dispersion, no-patch axons are more suitable to modulate large-scale dynamics of cortical synchronization.

## Data Availability Statement

The raw data supporting the conclusions of this article will be made available by the authors, without undue reservation.

## Ethics Statement

The animal study was reviewed and approved by Animal Care and Surgical Procedures were performed in compliance with NIH (National Institutes of Health) regulations and with the approval of the Institutional Animal Care and Use Committee of Vanderbilt University.

## Author Contributions

LN designed the study. YM, EP, and MA performed the research. YM, LN, and LZ made the data analysis. LN and YM wrote this paper. RF and AR were instrumental in the animal procedures and contributed to revisions of this manscript. All authors contributed to the article and approved the submitted version.

## Funding

This study was supported by NIH NS093998 and NKFIH-OTKA NN118902 as well as the Tempus Public Foundation Hungary under the Stipendium Hungaricum Scholarship.

## Conflict of Interest

The authors declare that the research was conducted in the absence of any commercial or financial relationships that could be construed as a potential conflict of interest.

## Publisher's Note

All claims expressed in this article are solely those of the authors and do not necessarily represent those of their affiliated organizations, or those of the publisher, the editors and the reviewers. Any product that may be evaluated in this article, or claim that may be made by its manufacturer, is not guaranteed or endorsed by the publisher.
