# Supplementary material for: Modular Organization of Signal Transmission in Primate Somatosensory Cortex
Source: Front Neuroanat. 2022 Jul 8;16:915238. doi: 10.3389/fnana.2022.915238 (PMC9305200; doi:10.3389/fnana.2022.915238)
Supplement: Supplementary file 1 [file Data_Sheet_1.PDF]

Supplementary Material

1 Supplementary Figures (Figure S1 –Figure S6)

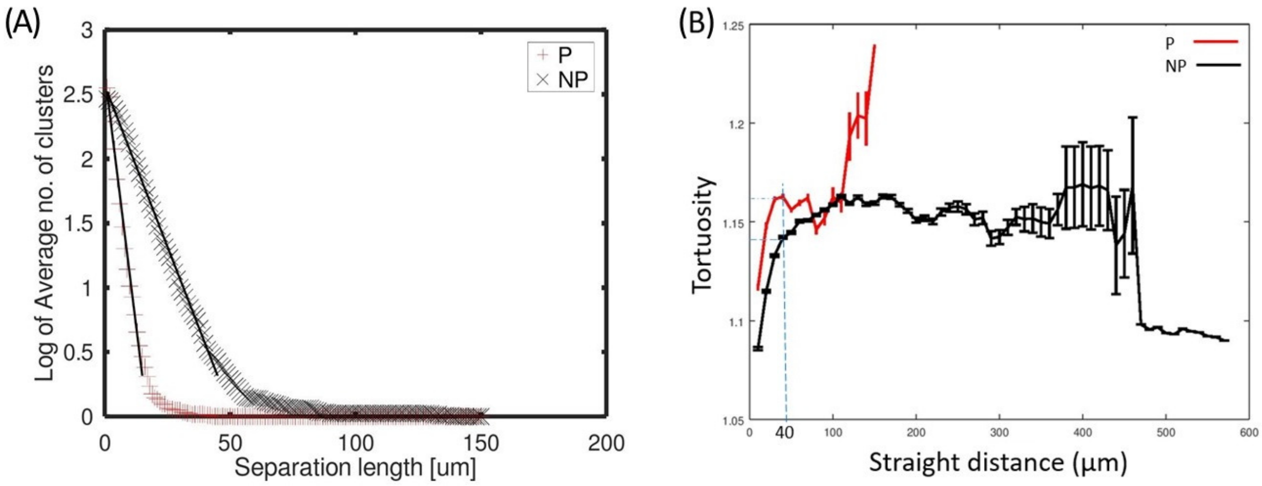

Figure S1. Determination of bouton clustering and tortuosity. (A) Sharply decreasing number of clusters as a function of increasing axon length. For the population of axon segments from the 6 cases, the linear fit has a slope of -0.16 (intersection: 2.67) for the patch axons and -0.05 (intersection: 2.57) for the no-patch axons. (B) A sigmoid-like increase of measured tortuosity resulting by an incremental 10 µm elongation of the axons from the start to the end point as determined from the population of patch and no-patch axons. Dashed blue lines show tortuosity values at the 40 µm length used in this study. Means and standard errors are shown. P: patch axons, NP: no-patch axons.

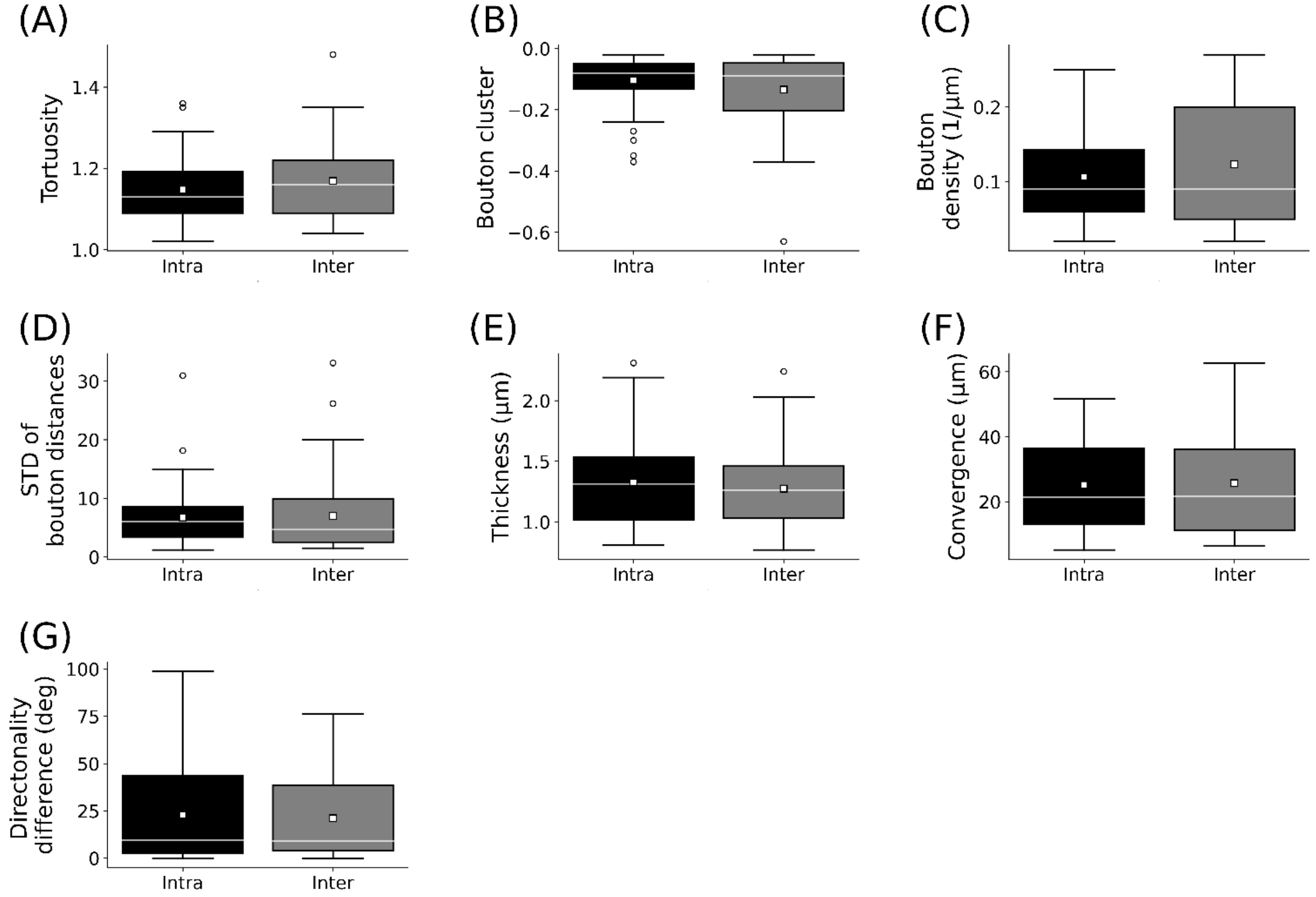

Figure S2. Comparison of variables between the intra-areal and inter-areal axons. Same conventions as Figure 3. Please see Tables 1 and 2 for the number of axons compared here and in the subsequent Figures.

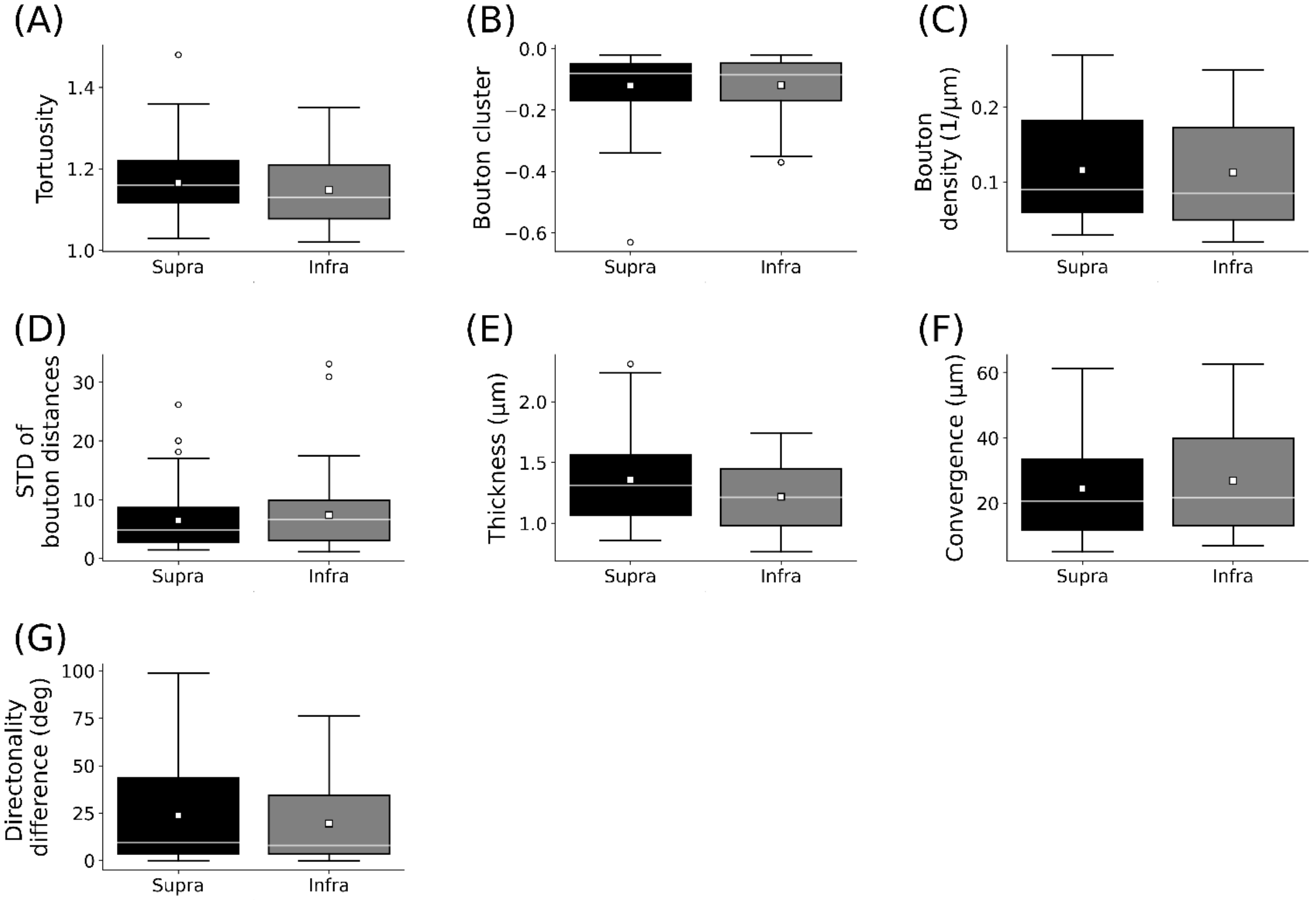

Figure S3. Comparison of variables between the supragranular and infragranular axons. Conventions are the same as Figure 3.

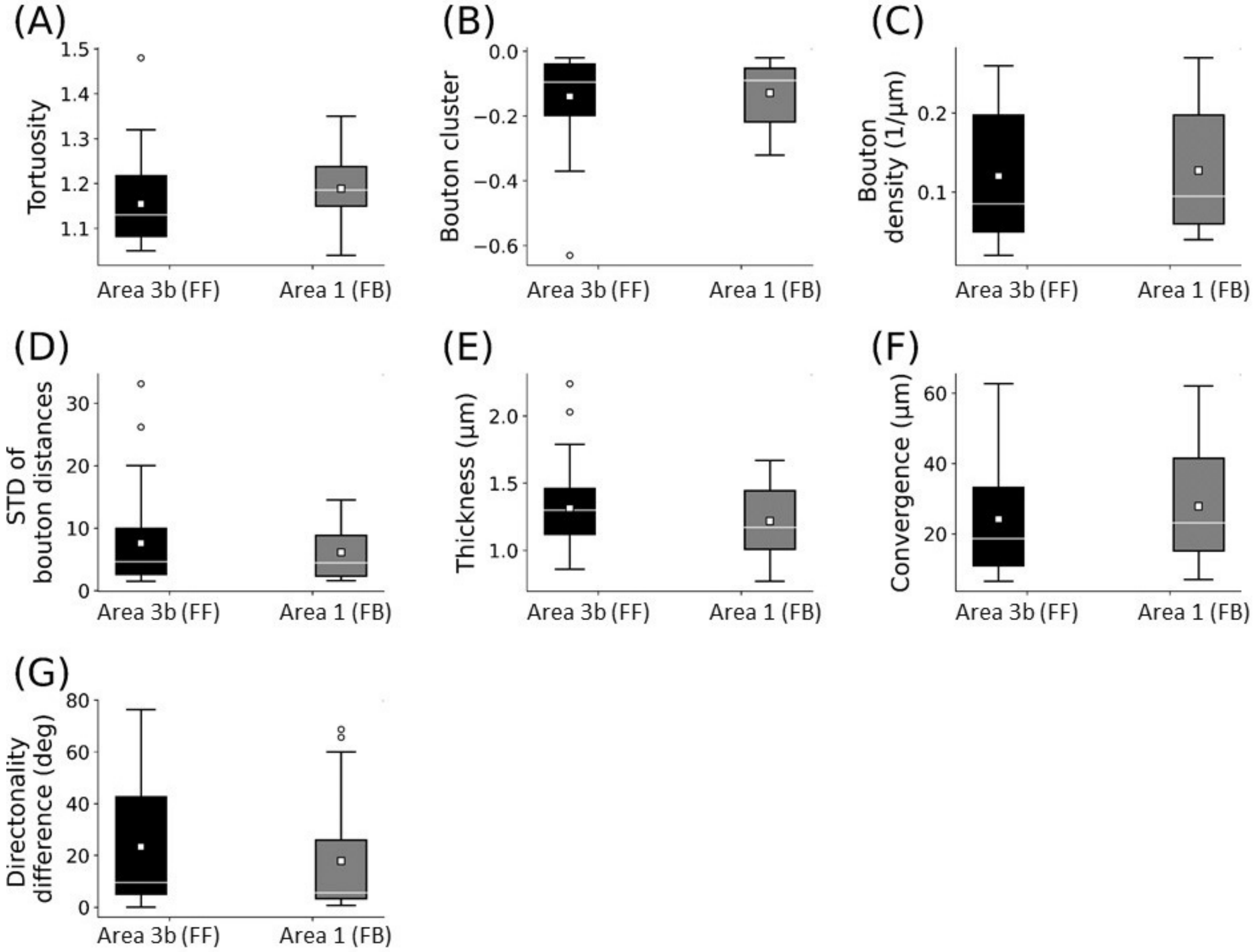

Figure S4. Comparison of variables between the feed-forward (FF) and feed-back (FB) inter-areal axons in area 3b and area 1. Conventions are the same as Figure 3.

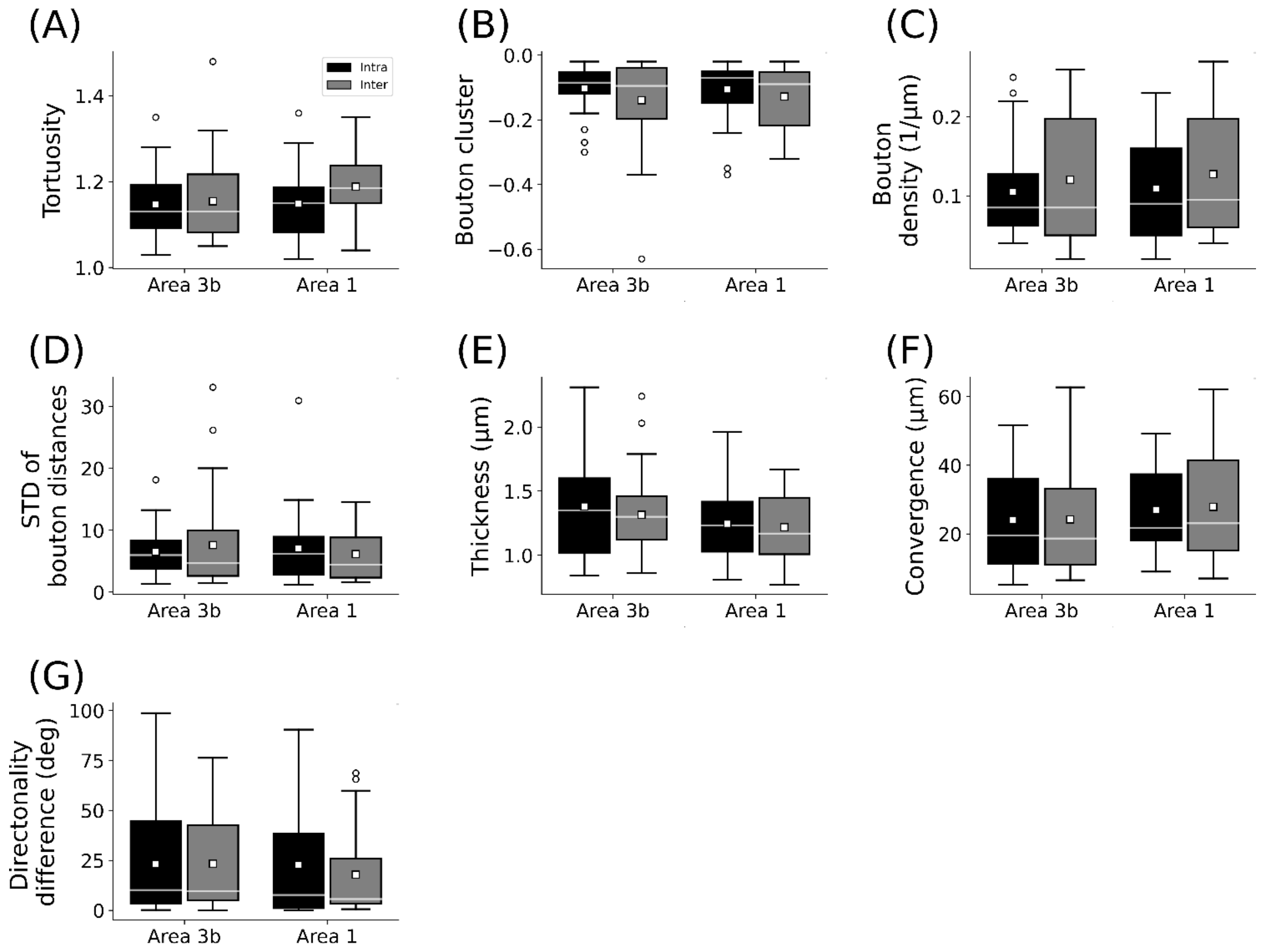

Figure S5. Comparison of variables within and between areas and injection sites: intra-areal and inter-areal axons. Conventions are the same as Figure 3.

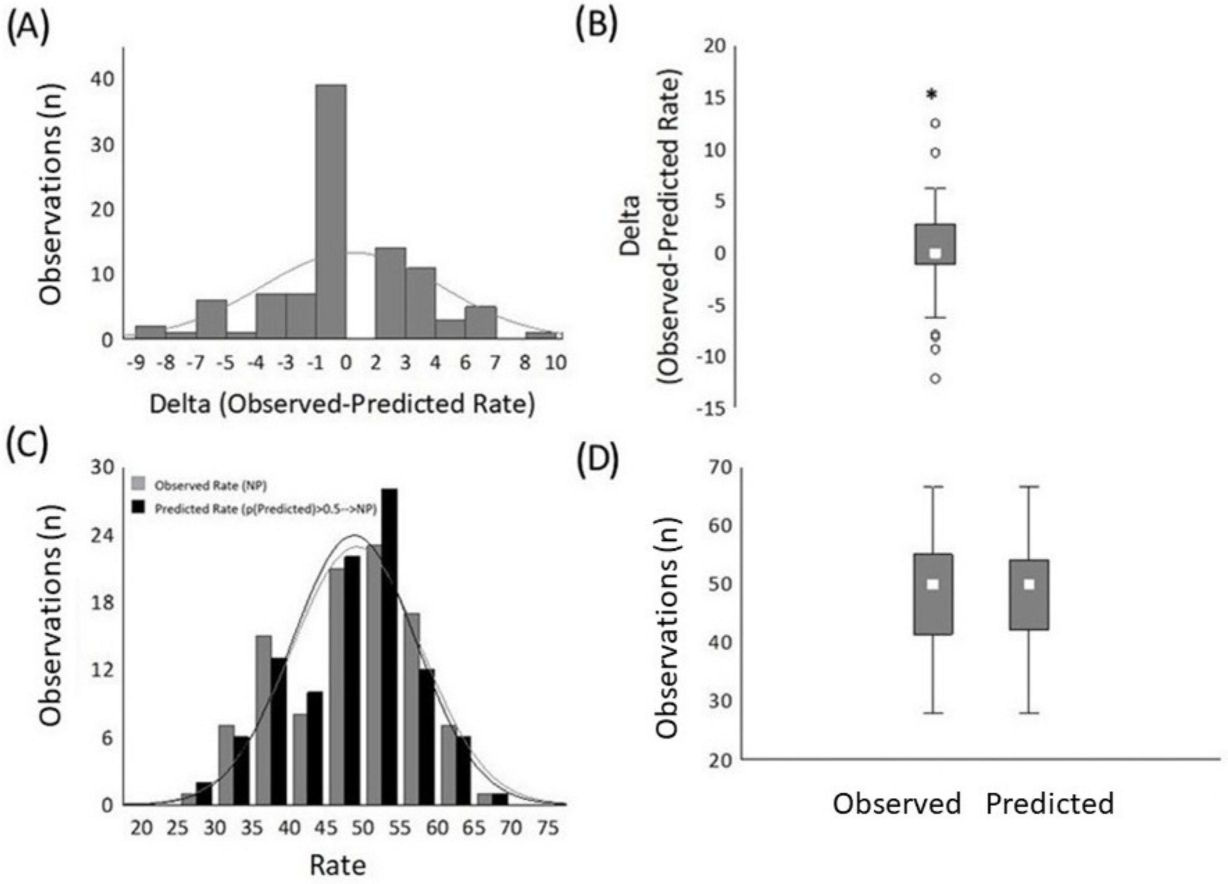

Figure S6. Validation of the stepwise logistic regression by bootstrap (100 replications, 25% holdout and 0.5 probability cutoff threshold). (A and B). Distribution (A) and mean +/- std (B) of the observed minus predicted rates in the full model. (C and D). Distribution (C) and mean +/- std (D) of the observed and predicted rates. Panels A-D show the negligible difference between the observed and predicted rates.

2 Supplementary Tables (Table S1 – Table S3)

Table S1. Sample size after averaging is shown by anatomical categories. Means of supra- and infragranular layers were obtained in the different cases. Laminar comparisons of P and NP axons has to be omitted due to the small number of cases. area 3b, area 1: injected area, Intra: intra-areal axon, Inter: inter-areal axon, FF: feed-forward axons, FB: feedback axons, supra: supragranular axons, infra: infragranular axons.

Total no	44
area 3b	24
area 1	20
P or NP	22
Intra or Inter	22
FF	12
FB	10
Supra	24
Infra	20
P-Intra or P-Inter	11
NP-Intra or NP-Inter	11

Table S2. Marginal table without Bouton-convergence

Effect	Somers' D	Estimate	Pr>Chi2	df
Tortuosity	0.9823	-0.3044	0.9657	1
Bouton cluster	0.9877	12.0204	0.1070	1
Bouton density	0.9888	-26.6115	0.0379	1
Bouton distance std	0.9842	0.1525	0.1719	1
Thickness	0.9996	31.0813	0.1538	1
Directionality difference	0.9985	-0.6328	0.0869	1

Table S3. Marginal table without Bouton-convergence and Bouton density

Effect	Somers' D	Estimate	Pr>Chi2	df
Tortuosity	0.9888	3.6100	0.6487	1
Bouton cluster	0.9896	2.9844	0.7651	1
Bouton distance std	0.9904	-0.3575	0.1954	1
Thickness	0.9996	108.5321	0.5725	1
Directionality difference	1.0000	-24.9717	0.4322	1
